# Determinants of quality of life in patients with fibromyalgia: A structural equation modeling approach

**DOI:** 10.1371/journal.pone.0171186

**Published:** 2017-02-03

**Authors:** Jeong-Won Lee, Kyung-Eun Lee, Dong-Jin Park, Seong-Ho Kim, Seong-Su Nah, Ji Hyun Lee, Seong-Kyu Kim, Yeon-Ah Lee, Seung-Jae Hong, Hyun-Sook Kim, Hye-Soon Lee, Hyoun Ah Kim, Chung-Il Joung, Sang-Hyon Kim, Shin-Seok Lee

**Affiliations:** 1 Division of Rheumatology, Department of Internal Medicine, Chonnam National University Hospital & Medical School, Gwangju, Korea; 2 Division of Rheumatology, Department of Internal Medicine, Inje University Haeundae Paik Hospital, Busan, Korea; 3 Division of Rheumatology, Department of Internal Medicine, Soonchunhyang University, College of Medicine, Cheonan, Korea; 4 Division of Rheumatology, Department of Internal Medicine, Maryknoll Medical Center, Busan, Korea; 5 Division of Rheumatology, Department of Internal Medicine, Catholic University of Daegu, School of Medicine, Daegu, Korea; 6 Division of Rheumatology, Department of Internal Medicine, School of Medicine, Kyung Hee University, Seoul, Korea; 7 Division of Rheumatology, Department of Internal Medicine, Soonchunhyang University Seoul Hospital, Seoul, Korea; 8 Division of Rheumatology, Department of Internal Medicine, Hanyang University College of Medicine and the Hospital for Rheumatic Diseases, Seoul, Korea; 9 Division of Rheumatology, Department of Internal Medicine, Ajou University Hospital, Ajou University School of Medicine, Suwon, Korea; 10 Division of Rheumatology, Department of Internal Medicine, Konyang University Medical School, Daejeon, Korea; 11 Division of Rheumatology, Department of Internal Medicine, School of Medicine, Keimyung University, Daegu, Korea; Stanford University School of Medicine, UNITED STATES

## Abstract

**Objective:**

Health-related quality of life (HRQOL) in patients with fibromyalgia (FM) is lower than in patients with other chronic diseases and the general population. Although various factors affect HRQOL, no study has examined a structural equation model of HRQOL as an outcome variable in FM patients. The present study assessed relationships among physical function, social factors, psychological factors, and HRQOL, and the effects of these variables on HRQOL in a hypothesized model using structural equation modeling (SEM).

**Methods:**

HRQOL was measured using SF-36, and the Fibromyalgia Impact Questionnaire (FIQ) was used to assess physical dysfunction. Social and psychological statuses were assessed using the Beck Depression Inventory (BDI), the State-Trait Anxiety Inventory (STAI), the Arthritis Self-Efficacy Scale (ASES), and the Social Support Scale. SEM analysis was used to test the structural relationships of the model using the AMOS software.

**Results:**

Of the 336 patients, 301 (89.6%) were women with an average age of 47.9±10.9 years. The SEM results supported the hypothesized structural model (χ^2^ = 2.336, df = 3, p = 0.506). The final model showed that Physical Component Summary (PCS) was directly related to self-efficacy and inversely related to FIQ, and that Mental Component Summary (MCS) was inversely related to FIQ, BDI, and STAI.

**Conclusions:**

In our model of FM patients, HRQOL was affected by physical, social, and psychological variables. In these patients, higher levels of physical function and self-efficacy can improve the PCS of HRQOL, while physical function, depression, and anxiety negatively affect the MCS of HRQOL.

## Introduction

Fibromyalgia (FM) is a relatively common syndrome, characterized by chronic widespread musculoskeletal pain, accompanied by various somatic and psychological symptoms [1]. The prevalence of FM has been estimated to range from 2% to 7% [2, 3]. FM patients describe diverse clinical manifestations, including fatigue, sleep disturbances, stiffness, skin tenderness, post-exertional pain, irritable bowel syndrome, cognitive disturbance, irritable bladder syndrome, headaches, dizziness, fluid retention, paresthesias, restless legs, and mood disturbances. These long-term somatic and psychological symptoms may lead to the deterioration of health-related quality of life (HRQOL).

HRQOL is an emerging issue in FM. Previous studies have compared FM patients with other subjects and have found that FM patients have a worse health status than patients with other chronic diseases, including osteoarthritis, rheumatoid arthritis, systemic lupus erythematosus, myocardial infarction, chronic obstructive pulmonary disease, congestive heart failure, hypertension, and diabetes, as well as healthy control subjects [4–10].

Several studies have investigated the impacts of socio-demographic, clinical, and psychological factors on HRQOL in patients with FM. In female FM patients, it was found that those patients who were employed reported a better state of health than those who were not [11, 12]. In a Spanish cohort, socio-demographic characteristics, such as the number of children, age, educational level, and concomitant rheumatologic diseases, showed an association with QOL [13]. A Turkish study investigated the relationships between QOL and a variety of variables, including socio-demographic factors, a visual analog scale (VAS) for pain, the Beck depression inventory (BDI), the tender points count (TPC), and the fibromyalgia impact questionnaire (FIQ). In this study, VAS and BDI scores were found to be negatively correlated with QOL; however, TPC did not correlate with QOL [14]. The influence of anxiety, self-efficacy, and social support on HRQOL has also been demonstrated in previous reports [13, 15–17].

However, no previous study has systematically investigated the relationship between HRQOL and numerous FM-related factors, including depression, anxiety, self-efficacy, and social support. Such a variety of variables enable a more reliable description of the results than single measures alone. This study is unique, therefore, in that it systemically investigated the relationship between HRQOL and the variables described above. Furthermore, previous studies have demonstrated such relationships through the use of only a simple correlation or a regression analysis method. The focus of regression analysis is on the relationships between a dependent variable and several independent variables. In that case, the model could expect to determine direct effects only from the independent variables to a dependent variable, rather than the indirect effects and interrelationships of the independent variables. In addition, the regression method can give misleading results regarding causality between variables. In the case of the higher-order factor model, multiple dependent variables exist; therefore, the regression method could not analyze all variables at one time.

Structural equation modeling (SEM), a unification of statistical techniques including path analysis, confirmatory factor analysis, and regression analysis, enables analysis of the interrelationships of independent variables and their indirect effects through other variables. SEM offers a more complex and diverse network of variables by including latent variables as well as observed ones. Thus, investigators can simultaneously analyze numerous factors affecting HRQOL using SEM. Because HRQOL is affected by various factors, SEM is an appropriate method for analysis.

Thus, in this study we used SEM for a comprehensive and persuasive analysis of HRQOL of FM patients for the first time. The present study assessed 1) relationships among physical function, social factors, psychological factors and HRQOL, and 2) the effects of these variables on HRQOL in a hypothesized model using a SEM approach.

## Materials and methods

### Study design

This observational study forms part of a prospective designed study, the ‘Korean Nationwide Fibromyalgia Epidemiologic Survey’ (KNFES), which is a well-characterized long-term follow-up cohort of fibromyalgia patients. KNFES investigates HRQOL and associated factors, such as demographics, socioeconomics, physical function, psychological function, and health status. The survey is scheduled to be followed up at 1-year intervals. It was conducted at 10 tertiary centers across the Republic of Korea and this research complied with the Helsinki Declaration. All subjects provided written informed consent at the time of enrollment.

The Institutional Review Board (IRB)/Ethics Committee at 6 each medical center approved the protocol for this study; the IRB of Chonnam National University Hospital (IRB number: 2010-03-058), the Ethics Committee Soonchunhyang University, College of Medicine, Cheonan, Korea (IRB number: SCHCA_IRB_2011–27), the IRB of Kyung Hee University Hospital (IRB number: 0850–08), the IRB of Daegu Catholic University Medical Center (IRB number: CR-09-089) the IRB of Maryknoll Medical center (IRB number: MMC.2008/8/14-5(43)).

This study was preceded by a cross-sectional analysis using the initial data from the KNFES. These data were collected between February 2008 and August 2009. To initiate our study, we first obtained a research consent form from each patient and then interviewed participants about their demographic and socioeconomic status using a systematic case report form. A physical examination was then performed by a trained rheumatologist and participants were asked to complete self-reporting questionnaires regarding HRQOL and associated variables. Finally, we analyzed the relationships among socio-demographic status, physical function, psychological factors, and HRQOL using SEM.

### Patient population

In total, 336 patients were registered for the KNFES. All of the patients were diagnosed with FM based on the 1990 American College of Rheumatology criteria [18], which were: 1) widespread pain in combination with 2) tenderness at 11 or more of 18 specific tender point sites. We included established FM patients as well as newly diagnosed ones. The patients had sufficient physical and psychological capacity to complete the self-reporting questionnaires. Patients who were illiterate or had limited vision received help from a trained clinical research nurse and rheumatologist. Among the 10 medical centers, some of the tertiary centers are located in metropolitan areas, while others are located in more rural areas. Thus, the distribution of patients according to region was generally even. Patients were administered medications, such as selective serotonin reuptake inhibitors (SSRI), serotonin-norepinephrine reuptake inhibitors (SNRI), pregabalin, gabapentin, tricyclic antidepressants, nonsteroidal anti-inflammatory drugs (NSAID), tramadol, acetaminophen, benzodiazepine, and muscle relaxants, depending on their symptoms.

### Variables

Demographic characteristics, including age, gender, duration of symptoms (years), years since the diagnosis of FM, alcohol intake, smoking status, body mass index (kg/m^2^), and underlying diseases (diabetes mellitus, hypertension, affective disorder and rheumatologic disorders), were assessed from a case report form and a review of the medical records. The patients’ socioeconomic status, such as number of years of education, marital status, and annual income, were also collected using the case report form. The duration of symptoms denotes the number of years from the time when FM symptoms (such as general weakness, poor sleep, fatigue, headache, esophageal dysmotility, irritable bowel syndrome, irritable bladder syndrome, and stiffness) first occurred. Alcohol intake and smoking status were identified at the time of enrollment into this study. Smoking status was classified as current smokers (smoked ≥ 100 cigarettes in their lifetime and currently a smoker) and non-current smokers (smoked < 100 cigarettes in their lifetime and not currently smoking). Alcohol consumption (within the past 12 months) was classified as current drinkers and non-current drinkers. Body mass index (BMI), body weight divided by the square of height (kg/m^2^), was calculated at the time of enrollment. Height was measured to the nearest 0.1 cm, and weight was recorded to the nearest 0.1 kg. We categorized patients as having an underlying disease in cases where medication had been administered. Patients’ annual income was calculated as the sum of the household income.

A physical examination was performed to assess the number and score of tender points at the time of enrollment. Examination of tender points was evaluated by a rheumatologist according to the standardized manual tender point survey [19]. The number of tender points was calculated by summing the count of tender points, and the tender point score was calculated as the sum of the score for each tender point: no pain = 0, tender when asked = 1, spontaneous verbal response = 2, and withdrawal = 3. Thus, the number of tender points ranged from 0 to 18, and the tender point scores ranged from 0 to 54.

To assess the physical/psychological variables and HRQOL, the FIQ, BDI, State-Trait Anxiety Inventory (STAI), Arthritis Self-Efficacy Scale (ASES), and Social Support and Short Form 36 (SF-36) health surveys were used. To evaluate physical function and health status, the Korean version of FIQ [20], which is a translated and validated version of FIQ, modified by us in 2002, was used. The Korean FIQ contains 10 items with a further 10 sub-items and the maximum score has been adjusted to 100. A higher score indicates a more negative impact. The BDI comprises 21 multiple-choice self-reporting questions for measuring the severity of depression [21]. Each question is assigned a value between 0 and 3 and the maximum total score is 63. STAI measures state anxiety (STAI-I, anxiety about an event) and trait anxiety (STAI-II, anxiety level as a personal characteristic) [22]. The two inventories of anxiety are composed of 20 items scored with a value from 1 to 4. Thus, the total score range is from 20 to 80. ASES was used to evaluate health behaviors such as physical activity, eating behaviors, and pain-coping strategies [23, 24]. It consists of three subscales: self-efficacy for pain management (3 items), self-efficacy for function (5 items), and self-efficacy for controlling other symptoms (6 items). Each item is scored from 1 (very uncertain) to 10 (very certain). Social family support was assessed to address the perception of social support adequacy from the family [25]. Finally, we used the Korean version of SF-36 v2 to assess HRQOL [26]. This is composed of eight scales, containing 2–10 items each and eight scales are grouped into two components of health status: a physical component summary and a mental component summary. The physical component summary (PCS) includes four scales, such as physical functioning, role-physical, bodily pain, and general health perceptions. The mental component summary (MCS) also includes four scales: vitality, role-emotional, social functioning, and mental health.

### Statistical analysis

Data are described as means ± standard deviations and numbers with percentage (%) of cases. The Pearson correlation coefficient was calculated to evaluate the relationships among the variables. Structural equation modeling, a type of multivariate analysis, was applied to evaluate the relationships among socio-demographic status, physical function, social and psychological factors, and HRQOL. Additionally, the effects of physical, psychological, and social influences on HRQOL were evaluated by Analysis of Moment Structure (AMOS; ver. 16.0). After regression analysis was completed, the total, direct, and indirect effects of variables to the PCS/MCS were calculated using the standardized regression weights for each pathway.

The model fit was tested to determine the “goodness-of-fit” between the hypothesized model and the data through the use of several methods; χ^2^ statistics and root-mean-squared error of approximation (RMSEA) were used as absolute fit measures. χ^2^ (*p* > 0.05) can suggest a good match between data and a hypothesized model. The ratio of χ^2^ to the degrees of freedom (df) value < 2 (χ^2^/df < 2) is also considered to be a good fit of the model. A RMSEA value < 0.05 is another indicator of a good fit. The normed fit index (NFI), relative fit index (RFI), incremental fit index (IFI), Tucker-Lewis index (TLI) and comparative fit index (CFI) were used as incremental fit measures. An incremental fit measure value > 0.9 indicated a ‘good’ fit for the model. Parsimonious normed-of-fit index (PNFI) and parsimonious comparative fit index (PCFI) were used as parsimonious fit measures. A value > 0.05 was considered reasonable for a good model fit.

## Results

### Sample characteristics

In total, 336 patients, aged between 13 and 75 years, with FM were investigated (S1 File). Of them, 301 (89.6%) were females with a mean age of 47.9 years. The average symptom duration was 8.31 years, and the average period of time since diagnosis of FM was 2.00 years. Of the patients, 81.3% were married, and the mean annual income was 32,940,000 Korean won (29,570 USD). About one-fourth of patients had an underlying disorder, such as an affective or rheumatologic disorder (26.5% and 26.2%, respectively). The mean number of tender points was 13.97and the tender point score was 27.03. The mean FIQ, BDI, STAI-I, STAI-II, Self-efficacy and Social Support scores were 59.73, 18.89, 49.03, 51.29, 729.10, and 38.43, respectively. The mean PCS and MCS SF-36 scores were 35.86 and 33.67, respectively (Table 1).

**Table 1:** Baseline demographic and clinical characteristics of 336 patients with fibromyalgia.

Age, years	47.94±10.86
Female (%)	301 (89.6)
Symptom duration, years	8.31±8.06
Disease duration, years	2.00±3.03
Alcohol (%)	105 (31.2)
Smoking (%)	48 (14.3)
Education, years	11.01±4.00
Marital status (%)	273 (81.3)
Annual income, million Korean won	32.94±32.15
thousand US dollars	29.57±28.86
Body mass index, kg/m^2^	22.82±3.39
Diabetes mellitus (%)	20 (6.0)
Hypertension (%)	59 (17.6)
Affective disorder (%)	89 (26.5)
Rheumatologic disorder (%)	88 (26.2)
Tender point, number	13.97±4.00
Tender point, score	27.03±13.35
FIQ	59.73±18.36
BDI	18.89±10.64
STAI-I	49.03±12.05
STAI-II	51.29±11.27
Self-efficacy	729.10±268.69
Social support-family	38.43±7.40
SF-36, physical component	35.86±7.49
SF-36, mental component	33.67±11.84

FM: fibromyalgia, FIQ: Fibromyalgia Impact Questionnaire, BDI: Beck Depression Inventory, STAI: State-Trait Anxiety Inventory, SF-36: Short-Form Health Survey.

Data are shown as means ± standard deviation.

### Correlation coefficients

Pearson correlation coefficients for the measured variables are presented in Table 2. FIQ was found to be directly correlated with BDI, STAI-I, and STAI-II and inversely correlated with Self-efficacy, Social Support, PCS, and MCS. BDI was directly correlated with STAI-I, and STAI-II and inversely correlated with Self-efficacy, Social Support, PCS, and MCS. STAI-I and STAI-II were found to be inversely correlated with Self-efficacy, Social Support, PCS, and MCS, respectively. Self-efficacy was directly correlated with Social Support, PCS, and MCS. Social Support was directly correlated with MCS. Finally, PCS was directly correlated with MCS.

**Table 2:** Correlations between structural parameters.

	FIQ	BDI	STAI-I	STAI-II	Self- efficacy	Social support	PCS	MCS
FIQ	1							
BDI	0.511**	1						
STAI-I	0.433**	0.671**	1					
STAI-II	0.430**	0.701**	0.688**	1				
Self-efficacy	-0.431**	-0.508**	-0.411**	-0.463**	1			
Social support	-0.146**	-0.264**	-0.247**	-0.243**	0.235**	1		
PCS	-0.475**	-0.294**	-0.229**	-0.207**	0.478**	0.064	1	
MCS	-0.587**	-0.630**	-0.564**	-0.600**	0.422**	0.208**	0.125*	1

*p < 0.05,

**p < 0.01.

FIQ: Fibromyalgia Impact Questionnaire, BDI: Beck Depression Inventory, STAI: State-Trait Anxiety Inventory, PCS: physical component summary, MCS: mental component summary.

### Structural equation modeling

The overall fit of our model was found to be acceptable. We obtained χ^2^ = 2.336, df = 3, χ^2^/df = 0.778, *p* = 0.506, RMSEA < 0.001 (90% CI = 0.000–0.084) as absolute fit measures, NFI = 0.998, RFI = 0.917, IFI = 0.947, TLI = 0.936, CFI = 1.000 as incremental fit measures, and PNFI = 0.083, PCFI = 0.083 as parsimonious fit measures.

From the regression analysis, FIQ and ASES were found to be related to the PCS of SF-36. For the MCS, FIQ, BDI, STAI-I, and STAI-II were significantly related. Within SF-36, the mental component showed a negative association with the physical component (Table 3).

**Table 3:** Regression weights between structural parameters.

	Nonstandardized estimate	Standardized estimate	SE	CR	*P* value
MCS ← FIQ	-0.195	-0.302	0.046	-4.233	< 0.0011***
MCS ← BDI	-0.268	-0.241	0.066	-4.035	< 0.0011***
MCS ← STAI-I	-0.114	-0.116	0.056	-2.044	0.041*
MCS ← STAI-II	-0.217	-0.207	0.061	-3.542	< 0.0011***
MCS ← Social support	0.016	0.010	0.064	0.257	0.797
PCS ← FIQ	-0.180	-0.440	0.030	-6.067	< 0.001***
PCS ← Self-efficacy	0.011	0.380	0.001	7.158	< 0.001***
PCS ← MCS	-0.139	-0.220	0.069	-2.005	0.045*
MCS ← PCS	0.064	0.041	0.195	0.329	0.742

*p < 0.05,

***p < 0.001

MCS: mental component summary, PCS: physical component summary, FIQ: Fibromyalgia Impact Questionnaire, BDI: Beck Depression Inventory, STAI: State-Trait Anxiety Inventory, SE: standard error, CR: critical ratio.

**Fig 1:**
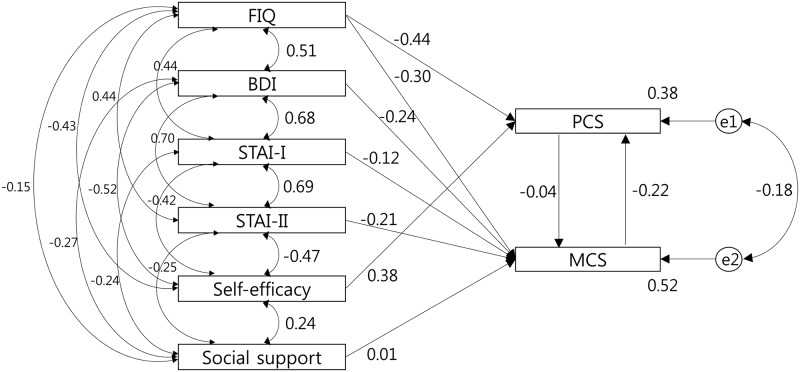
Significant pathways of the final model. FIQ: Fibromyalgia Impact Questionnaire, BDI: Beck Depression Inventory, STAI: State-Trait Anxiety Inventory, PCS: physical component summary, MCS: mental component summary.

Fig 1 and Table 4 illustrate the relationship between the structural parameters. The physical component can be seen to be directly related to Self-efficacy (β = 0.380, *p* < 0.001) and inversely related to FIQ (β = -0.440, *p* < 0.001). The mental component was inversely related to FIQ (β = -0.302, *p* < 0.001), BDI (β = -0.241, *p* < 0.001), STAI-I (β = -0.116, *p* = 0.041), and STAI-II (β = -0.207, *p* < 0.001).

**Table 4:** Total, direct, and indirect effects of independent variables on dependent variables.

	Effect	FIQ	BDI	STAI-I	STAI-II	Self -efficacy	Social support	PCS	MCS
PCS	Total	-0.370	0.053	0.025	0.045	0.377	-0.002	-0.009	-0.218
	Direct	-0.440	0.000	0.000	0.000	0.380	0.000	0.000	-0.220
	Indirect	0.070	0.053	0.025	0.045	-0.003	-0.002	-0.009	0.002
MCS	Total	-0.317	-0.239	-0.115	-0.206	0.015	0.010	0.040	-0.009
	Direct	-0.302	-0.241	-0.116	-0.207	0.000	0.010	0.041	0.000
	Indirect	-0.015	0.002	0.001	0.002	0.015	0.000	0.000	-0.009

PCS: physical component summary, MCS: mental component summary, FIQ: Fibromyalgia Impact Questionnaire, BDI: Beck Depression Inventory, STAI: State-Trait Anxiety Inventory.

Squared multiple correlations were 0.38 in the PCS and 0.52 in the MCS. Thus, our model accounted for 38% of the variance in physical QOL and 52% of the variance in mental QOL.

## Discussion

In this study, we provide a comprehensive model that illustrates the relationships between diverse variables and HRQOL in patients with FM. A higher FIQ score (i.e., poorer physical function, overall impact and disease severity) leads to a poorer physical component of HRQOL. However, higher self-efficacy, including physical activity, eating behaviors, and pain coping strategies, leads to a more favorable physical component. A higher FIQ score also has a direct negative effect on the mental component of HRQOL. Higher levels of depression and anxiety were found to be significantly negatively related to the mental component of HRQOL.

Our current study is of importance because we investigated several questionnaires related to QOL with a large number of FM patients, and established relationships among variables simultaneously using a SEM approach. To our knowledge, the methodology of SEM has not been applied before to the HRQOL of FM patients. Most previous studies on the HRQOL of FM used a correlation method to analyze the data from subscales of SF-36, unlike our study. Although this methodological difference can cause different results in this study, compared with previous studies, we believe that our model is unique in showing relationships to identify any effects on QOL in the complex network of QOL factors.

In this study, we found that FIQ and self-efficacy had a direct effect on the physical component of QOL. FIQ is well-known for its validated index, which reflects physical function and health status in FM. Birtane et al. showed that the total FIQ score correlated significantly with physical functioning, role-physical, and bodily pain of the physical component of SF-36 [4]. Tander et al. also reported a correlation between the total FIQ score and physical function, bodily pain, and general health of the physical component [14]. Although these studies were observational studies to investigate simple associations, FIQ seems to be associated with the physical component of QOL, and we were able to provide this association, apparently, by the SEM approach in this current study.

Regarding self-efficacy, several reports have demonstrated an association between self-efficacy and QOL. Borsbo et al. showed that higher self-efficacy was associated with better QOL, better general health, and less pain in chronic pain patients, including spinal cord injury-related pain, whiplash-associated disorders, and FM [27]. In a study by Burckhardt et al., the Quality of Life Scale was found to be positively associated with ASES [17]. These findings are in accordance with our results, describing an association between self-efficacy and the physical component of QOL. Self-efficacy is defined as “belief in one’s capability to organize and execute the courses of action required to manage prospective situations” and the factors affecting it have been identified as performance accomplishments, vicarious experiences, verbal persuasion, and physiological states [28]. According to this definition, comprehensive treatment programs regarding these factors are necessary to enhance self-efficacy in FM patients. Non-pharmacological and self-management strategies need to be combined with pharmacological treatment, to support self-efficacy and finally improve the HRQOL. In a meta-analysis of cognitive behavioral therapy (CBT) in FM patients, there was a significant effect on self-efficacy at post-treatment and at follow-up, although key symptoms of FM (pain, fatigue, and sleep disturbance) were not improved at post-treatment or at follow-up [29]. Thus, CBT can be considered as a non-pharmacological therapeutic option to improve self-efficacy in these patients.

In this study, we found that FIQ, BDI, and STAI had a direct effect on the mental component of QOL. In particular, the association between FIQ and the mental component in FM in the present study is noteworthy. In contrast with the association of FIQ with the physical component, previous studies on the effect of FIQ on the mental component are quite controversial. Although Birtane et al. [4] found no significant association between the total FIQ score and the mental component, Tander et al. [14] showed a negative correlation between the total FIQ score and only the social function and vitality sections of the mental component. Considering that both studies recruited only 30 patients to investigate this association and undertook analysis using the correlation coefficient between these variables, we believe that our SEM modeling provides a greater degree of solid evidence supporting the impact of FIQ on mental QOL. From a biopsychosocial perspective, the physical aspect of chronic pain can precede and lead to the development of a variety of mental health problems, such as depression and anxiety. Thus, it seems logical to say that chronic pain in FM can alter the physical functioning of these patients and physical dysfunction in FM can affect the mental aspects related to QOL. Our findings highlight the importance of multidisciplinary treatment, including pharmacological and non-pharmacological interventions, in these patients to reduce pain and improve QOL, which has also been shown to benefit patient outcomes [30].

We showed that depression and anxiety, as measured by the BDI and STAI, have a direct effect on the mental component of QOL. Given the epidemiological and pathophysiological similarities, it is unsurprising that recent data, including systematic reviews, indicate that depression, anxiety, and FM tend to occur as comorbid conditions [31]. Several studies have shown that depression and anxiety comorbidity predispose FM patients to lifestyles that adversely impact its clinical course, work capacity, and HRQOL [14, 32–34]. Considering that depression and anxiety appear as explanatory variables of the mental component of SF-36, the association of depression and anxiety with the mental component is relatively straightforward. The implication for clinicians managing FM patients is that we need to address psychological distress, by pharmacological treatments (e.g., duloxetine, milnacipran, and pregabalin), non-pharmacological interventions (e.g., exercise, CBT, and psychotherapy), or both [35]. Identifying factors that determine the extent of its impact on HRQOL will allow the design of more effective therapeutic strategies.

There are several limitations in the interpretation of our data. First, our observational study design is a limiting factor. Although our registry was designed for prospective research, our study is cross-sectional. Therefore, further longitudinal study is needed to establish causality. Second, because the surveys were conducted at tertiary centers, the patients’ living standards and their ability to access a tertiary center may have biased enrollment to favor patients with a more severe level of disease. Third, there was one 13-year-old patient in our sample; the other patients were all over 18 years old. When we analyzed the SEM excluding the 13-year-old, the results did not differ from the original final model. Fourth, medications were not quantified in our analysis. To enhance the parsimonious fit index, we could not include all of the parameters in our model. Consequently, medications might prove to be a considerable limitation of this study. Finally, FM patients with accompanying diseases, such as diabetes mellitus, hypertension, affective disorders, and rheumatologic disorders, are a confounding factor. Several reports have suggested that patients with diseases such as diabetes mellitus, hypertension, cardiovascular disease, rheumatoid arthritis, and ankylosing spondylitis showed lower QOL than healthy controls alone [36–38]. In our data, 185 patients had accompanying underlying diseases, 61 of whom had more than two comorbidities. Thus, the presence of comorbidities and their effects might prove to be a considerable limitation of this study.

In conclusion, HRQOL in FM patients was affected by physical, social, and psychological variables in our structural equation model. Higher physical function and higher self-efficacy may all improve the physical component of HRQOL, and better physical function and lower levels of depression and anxiety may directly improve the mental component of HRQOL. These findings underscore that physicians should initially evaluate physical function, activity, behaviors, coping strategies, and concurrent psychological illness, including depression and anxiety, by analyzing questionnaires related to these areas, as well as reviewing FM-related symptoms in the clinic.

## Supporting information

S1 File Supporting information exel file. Raw data on patients with fibromyalgia in this study.
(XLS)

## References

[1] Mease P. Fibromyalgia syndrome: review of clinical presentation, pathogenesis, outcome measures, and treatment. J Rheumatol Suppl. 2005;75:6–21. 16078356

[2] Jones GT, Atzeni F, Beasley M, Fluss E, Sarzi-Puttini P, Macfarlane GJ. The prevalence of fibromyalgia in the general population: a comparison of the American College of Rheumatology 1990, 2010, and modified 2010 classification criteria. Arthritis Rheumatol. 2015;67(2):568–75. 10.1002/art.38905 25323744

[3] Wolfe F, Ross K, Anderson J, Russell IJ, Hebert L. The prevalence and characteristics of fibromyalgia in the general population. Arthritis Rheum. 1995;38(1):19–28. 7818567 10.1002/art.1780380104

[4] Birtane M, Uzunca K, Tastekin N, Tuna H. The evaluation of quality of life in fibromyalgia syndrome: a comparison with rheumatoid arthritis by using SF-36 Health Survey. Clin Rheumatol. 2007;26(5):679–84. 10.1007/s10067-006-0359-2 16897118

[5] Burckhardt CS, Clark SR, Bennett RM. Fibromyalgia and quality of life: a comparative analysis. J Rheumatol. 1993;20(3):475–9. 8478854

[6] Da Costa D, Dobkin PL, Fitzcharles MA, Fortin PR, Beaulieu A, Zummer M, et al Determinants of health status in fibromyalgia: a comparative study with systemic lupus erythematosus. J Rheumatol. 2000;27(2):365–72. 10685798

[7] Hoffman DL, Dukes EM. The health status burden of people with fibromyalgia: a review of studies that assessed health status with the SF-36 or the SF-12. Int J Clin Pract. 2008;62(1):115–26. 10.1111/j.1742-1241.2007.01638.x 18039330 PMC2228371

[8] Kaplan RM, Schmidt SM, Cronan TA. Quality of well being in patients with fibromyalgia. J Rheumatol. 2000;27(3):785–9. 10743825

[9] Martinez JE, Barauna Filho IS, Kubokawa K, Pedreira IS, Machado LA, Cevasco G. Evaluation of the quality of life in Brazilian women with fibromyalgia, through the medical outcome survey 36 item short-form study. Disabil Rehabil. 2001;23(2):64–8. 11214717

[10] Salaffi F, Sarzi-Puttini P, Girolimetti R, Atzeni F, Gasparini S, Grassi W. Health-related quality of life in fibromyalgia patients: a comparison with rheumatoid arthritis patients and the general population using the SF-36 health survey. Clin Exp Rheumatol. 2009;27(5 Suppl 56):S67–74. 20074443

[11] Reisine S, Fifield J, Walsh SJ, Feinn R. Do employment and family work affect the health status of women with fibromyalgia? J Rheumatol. 2003;30(9):2045–53. 12966614

[12] Reisine S, Fifield J, Walsh SJ, Dauser D. Employment and quality of life outcomes among women with fibromyalgia compared to healthy controls. Women Health. 2004;39(4):1–19. 10.1300/J013v39n04_01 15691082

[13] Ubago Linares Mdel C, Ruiz-Perez I, Bermejo Perez MJ, Olry de Labry-Lima A, Hernandez-Torres E, Plazaola-Castano J. Analysis of the impact of fibromyalgia on quality of life: associated factors. Clin Rheumatol. 2008;27(5):613–9. 10.1007/s10067-007-0756-1 17909739

[14] Tander B, Cengiz K, Alayli G, Ilhanli I, Canbaz S, Canturk F. A comparative evaluation of health related quality of life and depression in patients with fibromyalgia syndrome and rheumatoid arthritis. Rheumatol Int. 2008;28(9):859–65. 10.1007/s00296-008-0551-6 18317770

[15] Pagano T, Matsutani LA, Ferreira EA, Marques AP, Pereira CA. Assessment of anxiety and quality of life in fibromyalgia patients. Sao Paulo Med J. 2004;122(6):252–8. 15692719 10.1590/S1516-31802004000600005 PMC11126175

[16] Schoofs N, Bambini D, Ronning P, Bielak E, Woehl J. Death of a lifestyle: the effects of social support and healthcare support on the quality of life of persons with fibromyalgia and/or chronic fatigue syndrome. Orthop Nurs. 2004;23(6):364–74. 15682879 10.1097/00006416-200411000-00005

[17] Alok R, Das SK, Agarwal GG, Tiwari SC, Salwahan L, Srivastava R. Problem-focused coping and self-efficacy as correlates of quality of life and severity of fibromyalgia in primary fibromyalgia patients. J Clin Rheumatol. 2014;20(6):314–6. 25160014 10.1097/RHU.0000000000000130

[18] Wolfe F, Smythe HA, Yunus MB, Bennett RM, Bombardier C, Goldenberg DL, et al The American College of Rheumatology 1990 Criteria for the Classification of Fibromyalgia. Report of the Multicenter Criteria Committee. Arthritis Rheum. 1990;33(2):160–72. 2306288 10.1002/art.1780330203

[19] Okifuji A, Turk DC, Sinclair JD, Starz TW, Marcus DA. A standardized manual tender point survey. I. Development and determination of a threshold point for the identification of positive tender points in fibromyalgia syndrome. J Rheumatol. 1997;24(2):377–83. 9035000

[20] Kim YA, Lee SS, Park K. Validation of a Korean version of the Fibromyalgia Impact Questionnaire. J Korean Med Sci. 2002;17(2):220–4. 10.3346/jkms.2002.17.2.220 11961307 PMC3054848

[21] Rhee MK, Lee YH, Park SH, Sohn CH, Chung YC, Hong SK, et al A standardization study of Beck Depression Inventory I—Korean version (K-BDI): Reliability and factor analysis. Kor J Psychopathol. 1995;4(1):77–95.

[22] Kim JT, Shin DK. A study based on the standardization of the STAI for Korea. The New Medical Journal. 1978;21(11):69–75.

[23] Buckelew SP, Huyser B, Hewett JE, Parker JC, Johnson JC, Conway R, et al Self-efficacy predicting outcome among fibromyalgia subjects. Arthritis Care Res. 1996;9(2):97–104. 8970267 10.1002/1529-0131(199604)9:2<97::aid-anr1790090205>3.0.co;2-f

[24] Lee HR, Park JS. The Influence of Self-efficacy on Activities of Daily Living in Patients with Rheumatoid Arthritis. J Korean Acad Adult Nurs. 2000;12(1):5–16.

[25] Oh KS, Oh KO, Lee SJ. Psychometric evaluation of the Korean Social Support Questionnaire. J Korean Acad Nurs. 2008;38(6):881–90. 10.4040/jkan.2008.38.6.881 19122490

[26] Han CW, Lee EJ, Iwaya T, Kataoka H, Kohzuki M. Development of the Korean version of Short-Form 36-Item Health Survey: health related QOL of healthy elderly people and elderly patients in Korea. Tohoku J Exp Med. 2004;203(3):189–94. 15240928 10.1620/tjem.203.189

[27] Borsbo B, Gerdle B, Peolsson M. Impact of the interaction between self-efficacy, symptoms and catastrophising on disability, quality of life and health in with chronic pain patients. Disabil Rehabil. 2010;32(17):1387–96. 10.3109/09638280903419269 20513205

[28] Bandura A. Self-efficacy: toward a unifying theory of behavior change. Psychol Rev. 1977;84(2):191–215. 847061 10.1037//0033-295x.84.2.191

[29] Bernardy K, Fuber N, Kollner V, Hauser W. Efficacy of cognitive-behavioral therapies in fibromyalgia syndrome—a systematic review and metaanalysis of randomized controlled trials. J Rheumatol. 2010;37(10):1991–2005. 10.3899/jrheum.100104 20682676

[30] Nuesch E, Hauser W, Bernardy K, Barth J, Juni P. Comparative efficacy of pharmacological and non-pharmacological interventions in fibromyalgia syndrome: network meta-analysis. Ann Rheum Dis. 2013;72(6):955–62. 10.1136/annrheumdis-2011-201249 22739992

[31] Pae CU, Luyten P, Marks DM, Han C, Park SH, Patkar AA, et al The relationship between fibromyalgia and major depressive disorder: a comprehensive review. Curr Med Res Opin. 2008;24(8):2359–71. 10.1185/03007990802288338 18606054

[32] Campos RP, Vazquez Rodriguez MI. Health-related quality of life in women with fibromyalgia: clinical and psychological factors associated. Clin Rheumatol. 2012;31(2):347–55. 10.1007/s10067-011-1870-7 21979445

[33] Consoli G, Marazziti D, Ciapparelli A, Bazzichi L, Massimetti G, Giacomelli C, et al The impact of mood, anxiety, and sleep disorders on fibromyalgia. Compr Psychiatry. 2012;53(7):962–7. 10.1016/j.comppsych.2012.03.008 22534032

[34] Kurtze N, Gundersen KT, Svebak S. Quality of life, functional disability and lifestyle among subgroups of fibromyalgia patients: the significance of anxiety and depression. Br J Med Psychol. 1999;72 (Pt 4):471–84. 10616131 10.1348/000711299160185

[35] Bernik M, Sampaio TP, Gandarela L. Fibromyalgia comorbid with anxiety disorders and depression: combined medical and psychological treatment. Curr Pain Headache Rep. 2013;17(9):358 10.1007/s11916-013-0358-3 23904203

[36] Chin YR, Lee IS, Lee HY. Effects of hypertension, diabetes, and/or cardiovascular disease on health-related quality of life in elderly Korean individuals: a population-based cross-sectional survey. Asian Nurs Res (Korean Soc Nurs Sci). 2014;8(4):267–73. Epub 2014/12/23. 25529909 10.1016/j.anr.2014.10.002

[37] Salaffi F, Carotti M, Gasparini S, Intorcia M, Grassi W. The health-related quality of life in rheumatoid arthritis, ankylosing spondylitis, and psoriatic arthritis: a comparison with a selected sample of healthy people. Health Qual Life Outcomes. 2009;7:25 10.1186/1477-7525-7-25 19296831 PMC2674445

[38] Rapaport MH, Clary C, Fayyad R, Endicott J. Quality-of-life impairment in depressive and anxiety disorders. Am J Psychiatry. 2005;162(6):1171–8. 10.1176/appi.ajp.162.6.1171 15930066

